# Maternal Behavior in Beef Cattle: The Physiology, Assessment and Future Directions—A Review

**DOI:** 10.3390/vetsci10010010

**Published:** 2022-12-24

**Authors:** Rory P. Nevard, Sameer D. Pant, John C. Broster, Scott T. Norman, Cyril P. Stephen

**Affiliations:** 1 School of Agricultural, Environmental and Veterinary Sciences, Charles Sturt University, Wagga Wagga, NSW 2678, Australia; 2 Gulbali Institute for Agriculture, Water and the Environment, Charles Sturt University, Wagga Wagga, NSW 2678, Australia; 3 Kallangur Veterinary Surgery, Kallangur, QLD 4503, Australia

**Keywords:** bovine, maternal, behavior, hormone, measuring, biomarker, calf performance

## Abstract

**Simple Summary:**

The general consensus on maternal behavior in free-ranging beef cattle was reviewed. Factors which may affect the display of these behaviors were also explored and provide important information relevant to calf survival and performance. A key aspect of the maternal instinct in all mammals relates to an intricate interplay of various hormones and neurotransmitters around the time of parturition. The influence of these hormones on regulating and initiating maternal behavior in a number of species were also described to provide unique insights for predicting maternal behavior in cattle. Lastly, current methods of measuring behavior were additionally summarized.

**Abstract:**

Bovine maternal behavior is known to be influenced by a variety of factors including hormonal mediation, breed, age, parity, host genetics and general management practices. Following centuries of varying levels of domestication processes, the behavior of the bovine cow has altered from that of her original wild ungulate ancestors, although many maternal instincts have remained unchanged. The influence of maternal behavior on calf health and performance is of interest to cow-calf beef production operations, as in most instances, the cow is solely responsible for rearing the calf until weaning. However, investigating the magnitude of this influence is challenging, in part because objective measurement of behavioral traits is difficult, particularly in extensive settings. In recent years, while a number of remote monitoring devices have been developed that afford opportunities for objective measurement of behavioral traits in livestock, characterization of physiological mechanisms that underlie superior maternal behavior, including identification of potential biomarkers remains elusive in cattle. Hormonal profiles during the periparturient period have been shown to influence behavioral patterns in both current and future generations in other mammalian species and may provide insights into the physiology of bovine maternal behavior. Therefore, the aim of this review is to describe general characteristics of bovine maternal behavior and the factors known to influence it, including hormonal drivers, through which cross-reference to other species is made. Current methods of measuring and assessing behavior that may also be applicable to most production settings have also been reviewed. At present, there is no known hormonal assay that can be used to measure and/or reliably predict bovine maternal behavior post-calving or across generations. Being able to objectively assess superior maternal behavior, whether that be through remote monitoring, hormonal profiling or indirectly through measuring calf performance will be beneficial to livestock industries in the future.

## 1. Introduction

Maternal behavior in most mammalian species has been defined as the pattern of behaviors exhibited by the mother that contribute to ensuring survival and health of the dependent offspring [1]. Maternal behavior in cattle has been defined by some authors as the cow’s care for the calf; which includes strong social interactions, milk provision and nursing, and protection from overt danger or predation [2,3]. These behaviors have been otherwise summarized as the cow’s willingness to devote her time, energy and resources for protecting and rearing her offspring [4]. Recently, the inclination of other cows to care for alien calves (‘crèching’) has also been anecdotally included in the definition of maternal behavior. Immediately prior, during and post-calving, other maternal behavioral patterns may include separation behavior, shelter seeking, nest building, parturition itself, cleaning the neonate, allowing suckling, and importantly; establishment of the cow-calf bond [5].

In beef production systems, successfully raising and nourishing a calf from birth to weaning is dependent on the success of a cow’s mothering ability [6]. The majority of calf deaths in rangeland environments such as those in North America have been found to occur within the first 24 h to 7 days of life [7]. During this first week immediately after calving, maternal behavior of the cow has been found to be an important determinant of neonatal survival [8,9]. Intervention strategies targeted at identifying or selecting for superior maternal behavior could therefore assist in improving calf survival attributable to poor mothering ability. Elsewhere in the world, such as rangeland environments in northern Australia, reproductive wastage and calf mortality continue to be key issues for extensive farming systems and can lead to significant reduction in productivity, economic profitability and contributes to poor animal welfare outcomes [10,11].

Mismothering and misadventure (Appendix A) has been suggested to account for a significant proportion of early fetal calf loss in some observational studies also performed in Australia [12,13]. Other recent work suggests that between 30–50% of calf losses may occur within a day to a week of calving, respectively, with stress and undernutrition of the pregnant cow being the major causative factors [14]. In these extensive northern Australian settings, dehydration of the calf appears to be major cause of death [15,16], likely due to failed milk delivery by the cow following nutritional deficiencies in late gestation and early lactation [17,18]. This reinforces the importance of achieving the adequate provision of nutrients to the newborn as part of successful mothering in the bovine. Maternal behavior of cattle and other ruminants has also been defined by the frequency and strength of dam-calf interactions. The number and frequency of interactions between cows and calves have been found to be an important determinant of neonatal survival in the early postpartum period [9,19]. This may hold promise for the use of objective measurements such as those afforded through remote monitoring devices to define a measurable behavioral proxy in extensive beef herds. The instigator of the behavioral patterns that occur prior to calving is most likely attributed to changes in hormones that occur leading up to parturition. These endocrine changes are crucial to the delivery of the fetus and later the bonding of cow and calf thereafter [3,20].

## 2. Prepartum Behavior

Cows in free-ranging herds are often reported to isolate from the herd and create a nesting site several hours before calving [5]. This has been reported both via subjective observation and by objective assessment of contacts measured via ultra-high frequency (UHF) proximity loggers [21]. It is noteworthy that compared to other ruminants such as sheep, cows show attraction to birth fluids up to 12 h prepartum, possibly suggesting that cow directed maternal behaviors are sensitive to olfactory cues prior to parturition [22]. The cow reportedly isolates herself in order to reduce interference from other females and calves which ensures the bond between dam and offspring can occur without disturbance [23,24]. Calving away from the herd has also been suggested to lower the risk of predation for the calf which is part of the ‘Hider/Follower’ paradigm described in wild ungulates by other authors [22,24,25]. While most cows deliver the fetus in lateral recumbency, they become increasingly restless as calving approaches, spending a majority of the preceding three hours in a standing position [26]. The time spent standing could be either attributable to vigilance against predators or pain and anxiety associated with the onset of uterine contractions in Stage 1 of labor [27]. In summary, the goal of these behaviors around the time of calving by the cow is to ensure both successful physical delivery of the fetus as well as ensuring an optimal environment for the onset of postpartum maternal behaviors. This is achieved by firstly locating an appropriate birth site with low risk of predation, disturbance, and offspring mistaken identity [22]. Successful completion of the described prepartum behaviors ensures postpartum success and potentiates offspring survival [22].

## 3. Postpartum Behavior

**Figure 1:**
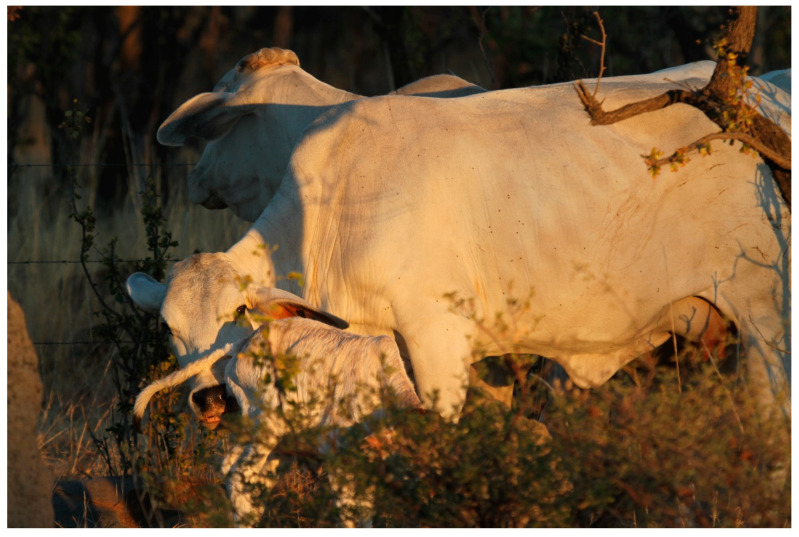
Example of typical postpartum behavior exhibited by a *Bos indicus* beef cow shortly after parturition. A matriarchal herdmate who is about to deliver her own calf stands close-by on high alert.

Cows bond almost immediately with their calves in the postpartum period and this is believed to be triggered by both the amniotic fluid from within the fetal membranes and hormonal changes within the dam herself [19,28]. Immediately after calving, the cow will devote the majority of her time licking and sniffing the calf, possibly to stimulate calf activity, facilitate drying from maternal uterine fluids to reduce heat loss, as well as socially bonding with the calf [29]. The licking behavior of cows is also believed to stimulate urination and defaecation by the calf, and the mother does this by repeatedly licking the ano-genital region of the calf [30]. Figure 1 shows a Brahman cow licking her calf whilst also directing toward her udder.

Licking and sniffing behaviors are a well-recognized component of the formation of the cow-calf bond which is crucial to ensuring ongoing survival, health and performance of the calf up until weaning [3]. Intensity of licking behavior peaks in the first hour after calving and then declines thereafter [25]. Interestingly, in Friesian dairy cattle, multiparous cows have been observed to exhibit higher intensities of licking during the first hour post-calving compared to first-calf heifers, whereas heifers were observed to exhibit a higher licking intensity in the second hour compared to multiparous cows [29]. This suggests that inexperienced mothers may take longer to initiate interactions with her calf. Nonetheless, there is agreement within the literature that this maternal bond is established and cemented by cow-calf pairs very early following parturition. This theory was supported by [31], in which two experiments were conducted with the aim of understanding the development of the early relationship between the cow and calf. In the first experiment, calves were left with their own mothers for either 24 or 96 h after birth. After only 24 h of interaction, it was found that each cow could discriminate between her calf and similar alien calves, however if cows were taken away at 24–96 h and re-joined with a different calf three days later, cows were unable to make a distinction between their own calves and alien calves suggesting three days separation is enough to break down the maternal bond [31]. The second experiment compared cow behaviors on subsequent calf re-introduction for those who either had their calf removed immediately or were allowed early calf contact for several hours. The cows that had calves removed immediately exhibited significantly lower interest in their own (or alien) calves on reintroduction, compared to those who had interaction time. In summary, the cow must develop a bond with her calf immediately postpartum to develop recognition and be able to discriminate alien calves [31]. These calf separation experiments have also been performed by other authors, and show similar findings [32]. Behaviors such as close contact, licking and suckling are crucial to this bond development [26]. Remote monitoring devices such as proximity loggers and pedometers may be able to objectively assess trends in cow-calf contact and suckling bouts, however recording the nature of early contact behavior may still entail a degree of subjectivity.

## 4. Interest in Fetal Membranes

**Figure 2:**
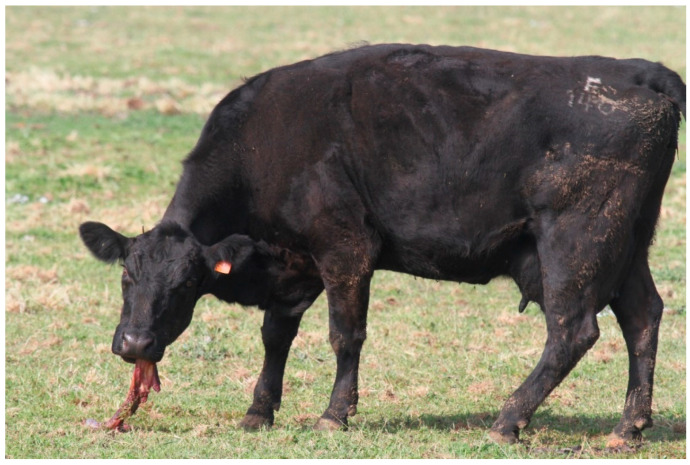
Postpartum cow exhibiting placentophagia behavior.

Other postpartum behaviors displayed by the cow such as licking also include ingesting the fetal membranes and exhibiting the flehmen response when licking and interacting with amniotic fluids on the calf [29]. It has been suggested that cows ingest fetal membranes to deter predators [5], or possibly because ingestion of amniotic fluid and placental tissue may afford analgesic-like effects [33,34]. Outside of the window around calving, cows do not seem to show the same level of attraction to these tissues and fluids, and it has been reported to only last from 12 h before-, to 24 h-post calving [35]. The interaction with the amniotic fluids is important, as it may be used as an olfactory cue for the mother to recognize her offspring and help enable the bond formation [36]. Figure 2 provides an example of a cow ingesting her own fetal membranes, otherwise known as ‘placentophagia’.

## 5. Contacts and Behavior in the Postpartum Period

To facilitate the aforementioned crucial cow-calf bonding behaviors of licking, nurturing and suckling, close contact between the cow and her calf must occur. Close contacts between cows and calves immediately after birth is essential to the formation of the maternal bond and calf survival [37]. This is reiterated as animal contact is a measurable trait that can be recorded by remote monitoring devices in extensive environments and potentially holds promise for achieving objectivity in behavioral assessment [38]. Intensive and intimate contact in the sensitive period immediately after calving is firstly facilitated by selection of an undisturbed calving environment by the mother [22]. For cows who isolate themselves from the herd for calving, the cow-calf pair will then re-join the herd within days which highlights that these ungulates portray both ‘hider’ and ‘follower’ type behavioral tendencies in herd settings [22]. Proximity between the cow and calf is initially maintained by the mother for the first few hours of life, and this distance starts to increase after several hours as the cow begins to recommence grazing following parturition [29]. As the calf becomes more agile and gains mobility, this distance then reduces, suggesting that both the cow and the calf have a role in maintaining proximity [39]. Using contact logging devices, it was also found that cows exhibited significantly higher contacts and longer duration of contacts with their own calves compared to the rest of the herd from calving until weaning [40]. This close contact is firstly facilitated by the cow, and then maintained by both cow and calf until weaning when the duration and frequency of interactions then gradually reduce over time [9]. The proximity between cow and calf is essential to not only allow feeding but also in maintaining social interactions, grooming, resting and predation prevention, allowing the social bond to develop further [39,40]. This close proximity during early life has also been shown to be important for subsequent maternal behavior of that offspring [20,32]. This may further suggest inter- and transgenerational effects of early contact and maternal behavior.

**Figure 3:**
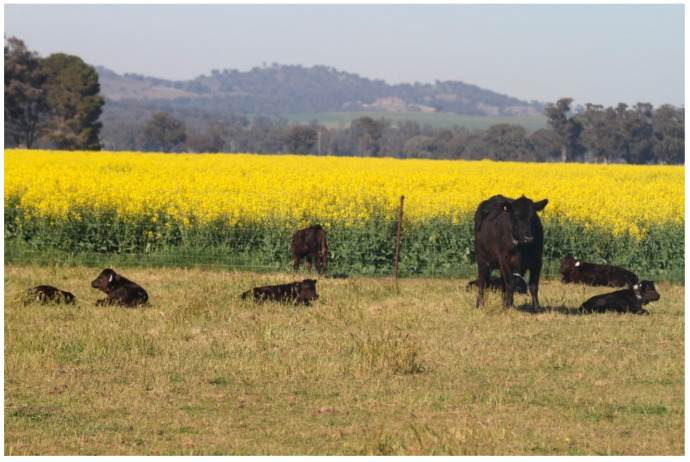
Example of a ‘crèche’ formed by a mature Angus beef cow.

Another characterized aspect of bovine maternal behavior is the formation of what is known as a ‘crèche’ whereby a number of offspring are watched over and protected by a single cow, whether the calves are her own or another cow’s offspring [41]. Anecdotal reports also exist from observations in extensive production systems in Australia whereby individual cows will form these ‘crèches’ whereby a single or secondary cow may oversee a group of calves for other mothers, suggesting a cooperative role in mothering in the postpartum period [42]. Whilst the role of these ‘crèches’ (Figure 3) is not completely understood, it could identify mothers that may contribute to the holistic well-being and production of the herd by allowing other herdmates to graze whilst the calves are monitored and protected from predation by that said individual [42]. 

In summary, close proximity in the immediate postpartum period is important not only to facilitate nursing, nutrient supply, and predation protection which are critical to the health of the calf, but also for social and behavioral reasons, which may have transgenerational effects. Consequently, a number of studies have attempted to investigate these behaviors either via direct observation or via remote monitoring devices (Table 1).

## 6. Maternal Contact and Calf Health

The effects of maternal behavior on calf health, survival and performance have been widely investigated. There is extensive literature focused on the impact of early cow-calf contacts on ongoing calf welfare, survival and performance although this work mostly pertains to the dairy industry where it is common practice to separate cows and calves immediately [55,56]. Recent systematic reviews in dairy cattle summarised the long-term effects of either early calf separation on cow/calf health [57] or prolonged cow-calf contact on calf behavior, welfare and sociability. Most studies investigating extended contact on social behaviour reported beneficial findings [56]. Some of the reported effects of prolonged contact included: increased licking and suckling of calves by cows who had themselves been mothered [58]; more sniffing and mock fighting by older calves in social tests [59]; and faster habituation by calves on group reintroduction [60]. While this current review focuses on beef cattle, dairy cattle studies which investigate the long-term effects of calf separation provide important insights into the impact of cow-calf contacts in early life. Most of the dairy cattle studies reviewed found that extended cow-calf contact was beneficial to long-term social interactions, reduced abnormal behaviors and importantly, learned mothering ability.

Besides the impact on social aspects various studies have also reported the impact of extended cow-calf contact on performance traits such as calf growth [56]. Of the 22 studies included in this systematic review which focused mostly on dairy cattle, 14 showed increased calf growth during the suckling period in calves that had extended contact with cows. However, the increase in calf growth in these studies was likely a reflection of increased milk intake as opposed to simply the influence of social contact, and the studies in which feed intake was restricted, but social cow contact was promoted showed no effects of contact on growth [56]. Similarly mixed findings have been reported in the albeit scarce beef cattle/dual purpose breed studies that exist [61,62,63]. A particular study of interest in beef cows compared the nature of maternal behavior exhibited by the cow on calf growth however provided confusing findings [64]. By assigning an arbitrary Maternal Aggression (MA) or Mothering Aptitude Score (MOM) assigned to the cow during calf handling and marking, scoring systems based on behavioral characteristics of beef cows at calving were able to be compared to subsequent weaning weights of calves. From a considerable sample size used in the experiment, it was found that calves born to cows with lower MA scores (less aggression) were lighter than those with a higher MA score (more aggressive) [64]. However, somewhat counterintuitively, the cows with the highest MA and MOM scores together had calves with the lightest weaning weight. Either excessive cow aggression or incongruency between behavioral assessments may have contributed to these conflicting results. Nonetheless, it suggests that maternal behavior at calving does affect performance at subsequent weaning, and this could possibly be related to milk provisioning and delivery [64].

## 7. Suckling and Immunity

Milk provision and nursing is an important aspect of maternal behavior in postpartum cows, and is crucial to both the survival and success of the newborn [2]. After the bond between cow and calf are developed immediately after birth, it is reinforced thereafter through suckling events. Cows will allow suckling by their calves and this is initiated by the cow within the first few weeks of life and then maintained by both cow and calf thereafter [25]. Recent observational work found that calves take time to begin nursing properly as it may take around three days postpartum for individual calves to develop a consistent nursing pattern [65]. The supply of milk for the newborn by the cow is critical to the transfer of immunity via immunoglobulins found in colostrum around the time of parturition. Calves are born without these circulating immunoglobulins (otherwise known as agammaglobulinemic), and they depend on the passive transfer of these immunoglobulins from maternal colostrum provided by the cow’s milk in the first few hours of life [66]. This reliance on the cows’ milk for the supply of early immune factors is a result of the epitheliochorial placentation of ruminants which does not allow the transfer of large molecules such as immunoglobulin G (IgG) between the dam and fetus during pregnancy [67]. A failure of the calf to absorb sufficient immunoglobulins from colostrum in the first few days of life is known as ‘failed transfer of passive immunity’ (FTPI), and this results in a serum IgG concentration less than 10 g/L in calves aged 24 h or more in dairy calves [68]. It is generally accepted that IgG < 10 g/L indicates failure of transfer of passive immunity (FTPI) in beef calves, and IgG of >16 and >24 g/L are associated with decreased morbidity and mortality [69]. Some authors will also recommend >24 g/L to increase the chances of lifelong survival [70]. A failure of passive transfer of immunity to the calf is associated with increased calf mortality and morbidity, as well as decreased health and longevity [71]. More specifically, FTPI can lead to an increased severity of diarrhoea, respiratory disease, increased shedding of pathogens and neonatal sepsis [68]. The longer term benefits of adequate passive transfer of immunity may also entail increased daily gain, feed efficiency, fertility and subsequent milk production of the calf when mature [67]. The successful transfer of these immune factors to the calf is multifactorial. The amount of immunoglobulins a calf absorbs is firstly related to the amount of immunoglobulins in colostrum, i.e., colostrum quality, secondly the amount of colostrum ingested by the calf itself, and thirdly the timing of ingestion by the calf [68]. Importantly, the volume of colostrum a calf ingests depends on its own strength and mobility, as well as udder conformation and mothering instincts of the dam [72]. Whilst the cow may require a degree of responsibility in ensuring adequate passive transfer, it is also important to note that the general vigor of the newborn is also critical to the adequate absorption of immunoglobulins from colostrum [73]. An interesting study found that calving cows in a group pen as opposed to an open paddock was related to increased risk of reduced colostrum intake by the calf, and this was a result of interference of the important cow-calf bond and mismothering [74]. Others have also reported the incidence of ‘allosuckling’ whereby calves suckle from alien cows not their own mother, and this has been reported to be more frequent in cows which calve in group calving facilities [75]. This could be a sequalae that occurs due to interference of the cow-calf bond formation, as calves are less likely to suckle alien cows when cow-calf pairs are provided access to secluded areas [76]. Further, allosuckling is also reported to be of lower incidence in *Bos indicus* type animals [77] which may suggest a decreased likelihood of cooperation in these breeds. However, as others have suggested [78], the unique patterns of seclusion behavior between individuals at the time of calving implicates that further work is needed to elucidate on the effects of interference and breed on cooperative milk provisioning. Failed milk delivery has also been identified by previous authors to be a crucial determinant to calf survival in extensive environments in northern Australia [14,15,17]. Whilst this is most likely a reflection of nutritional stressors in late gestation cows and heifers, it ultimately highlights that supply of milk, nutrients and immunity is a crucial aspect of successful mothering by the bovine cow.

## 8. Factors Influencing Maternal Behavior

Any behavioral pattern that may jeopardize the bond between mother and offspring has been classed as abnormal, and typically includes abandonment, failure to groom, aggression, delay in milk provisioning, or failure to allow suckling [79]. Some factors which have been known to influence maternal behavior are the breed, parity, cow body condition, calf sex as well as calf birth-weight [2,80]. Besides factors attributable to health, social interactions of the cow and calf within larger animal groups is complex and varies with calf age, parity and strength of cow-calf bond [81]. Although in general, observational studies in sheep and cattle have shown that successful maternal investment in neonatal survival is exemplified by longer and more frequent intimate social interactions [25,39,50,82,83]. It is also known that hormonal changes during the periparturient period have been shown to regulate specific aspects of maternal behavior in other mammalian models and there is relatively little research available on this topic in the bovine [26]. Maternal behavior in ruminants, in general, is initiated and regulated by hormones around the time of parturition, and differences or deficiencies between individuals may account for the variability in behavioural expressions [20]. Previous studies have attempted to identify a number of physiological biomarkers for bovine maternal behavior through the use of measurements of cow saliva cortisone and oxytocin, as well as optic thermal imaging and physiological parameters such as heart rate and observed number of interactions between cow and calf [2]. However, these approaches have had limited success. Other authors have also reported a degree of genetic control for maternal behavior [84,85,86], with a main limitation being identifying a selectable phenotype for links between certain gene sequences and superior behavior [3,23,53]. The genetic links to maternal behavior suggests that there may be an opportunity to potentially identify novel phenotypic traits in cattle with superior maternal behavior to reduce calf mortality. Blood hormonal profiles may offer a promising opportunity to assist in identifying superior mothers and contribute to the generation of these potentially selectable traits.

### 8.1. Breed

For many years, dairy calves have been removed from their mother and artificially raised which has reduced the selection pressure for maternal care in these breeds [56]. Contrary to this, beef breeds have mostly been reared under extensive circumstances meaning that the successful rearing of the calf is almost totally dependent on the cow’s own investment [26,58]. Previous studies indicate that there are significant differences between certain subspecies and breeds of the ‘Bos’ genera with expression of maternal behavioral characteristics [5,26]. It has been reported that Angus cows (beef) and Simmental (dual-purpose) cows responded differently to human handling of newborn calves with the Angus cows being more attentive and aggressive in their protection of the calf [51]. Others have also reported that the beef cows were more defensive of their calves in response to handling and tying up of the calf compared to other dairy breeds [2]. This accords with a previous study conducted in France involving Friesian (dairy) and Salers (beef) cows that demonstrated that beef cows had greater interaction with other animals of the herd and had a stronger attachment to their young compared to the dairy cows [53]. It has been hypothesized that this relationship is likely a consequence of many years of selective breeding for specific management practices, for instance dairy cows who have had historically their calves removed on the day of calving and beef cows which generally rear their own calves until weaning [26,51]. Similar to the aforementioned observational studies, contact between straight Brahman cows measured using radio-telemetric data from UHF proximity logger collars was indeed different to that recorded by the Belmont Red cows (which are a mix of both *Bos indicus* and *Bos taurus* breeds) [45]. Breed differences have also been found in other ruminant species such as sheep, where it was found that lowland breeds (Suffolk) consistently showed poorer maternal behavior compared to hill breeds (Scottish Blackface), which was characterized by a higher frequency of rejection behaviors, less time spent grooming, less cooperation to suckling by the offspring, and less vocalizations [87]. It was hypothesized that the differences in behaviours between these sheep breeds could be a reflection of underlying variations in hormones and individual maternal physiology [87]. Whilst breed differences are well reported, it is also worth noting that there is a known variation in the expression of maternal behavior between individual cows themselves. Individual differences in protection, aggression and vocalization behaviors when approached by a vehicle have been previously reported [88]. This suggests not only an inter-species and breed variation, but also individual variation in temperament and maternal vigilance. This variation between individuals may suggest the possibility that maternal behaviour should be able to be influenced by genetic selection pressure. As mentioned, suckling behavior, which is another critical aspect of maternal behavior has also been reported to vary between breeds. Some authors have found that there are notable differences between certain breeds with respect to the number and duration of suckling bouts of cow-calf pairs [43]. For instance, it was found that beef cow-calf pairs had a longer duration of suckling compared to the dairy cows [58]. Using both visual observations and video recordings, it was also found that when comparing Zebu (*Bos indicus*) cows and crossbred (*Bos taurus* × *Bos indicus*) cows, the zebu cows had a longer duration of contact whilst allowing suckling by their own calves and showed more agonistic actions against alien calves compared to the crossbred animals [54]. Latency to nurse has been reported to be longer in dairy breeds (two-six hours) as opposed to one hour reported in beef breeds which may suggest that beef breeds are more motivated to allowing suckling [26]. Again, this is likely a reflection of years of domestication and selection pressures placed on different animals. The differences between breeds in not only the nature of cow-calf contact, but also the variation in suckling behavior raises further avenues of research pertaining to milk provisioning and nursing in superior cows.

### 8.2. Age and Parity

Previous studies indicate that the prevalence of mismothering declines with parity, possibly due to learned maternal instinct fostered over subsequent generations [12,89,90]. The increase in mismothering accounts have been anecdotally attributed to a lack of experience in successfully raising calves by first calf heifers as opposed to older cows [89]. Interestingly, one author also reported a higher incidence of calf loss in cows with a previous history of unsuccessful calf rearing, indicating repeated episodes of mismothering in some cows [89]. An observational study which was performed on the Barkly Tablelands in the Northern Territory in Australia, found that first calf heifers would often ‘lose’ their calf when attending a watering point [13]. This is also in agreement to previous observational studies which concluded that primiparous dams showed a higher incidence of abnormal behavior, characterized by observational measures such as maintenance of proximity, contacts, licking and placentophagia [2,29]. In another study conducted with Belgian Blue *Bos taurus* cows which had calves delivered via caesarean section, it was found that the mature cows were considered better mothers than first calf heifers [91]. The differences observed between cows and heifers in this scenario is possibly a consequence of a combination of both previous vaginal delivery paired with experience, as other studies have demonstrated an effect of epidural anesthesia on hormonal interactions, namely via oxytocin, at the time of parturition [92,93]. Studies in other livestock have shown that mothering ability is also influenced by their experience as a neonate. It was suggested that the ability of mothers to display well-adapted maternal behavior is modulated by their own maternal experience from neonatal life and at first parturition [20]. In an intensive observational study in Friesian dairy cows, it was shown that close proximity between cow-calf pairs was predominantly maintained by the mother during the first three calvings of her lifetime, although maintenance of contact became less driven by solely the cow in the following calvings [29]. Primiparous cows also showed a more rapid decline in maternal responsiveness when reunited with their calf after a period of separation, than that of multiparous cows [31]. Whilst the differences between heifers and older cows could be simply a reflection of experience, others have hypothesized that differences in maternal behavior according to parity could also be attributed to changes in certain hormones such as oxytocin [94].Cumulatively, current knowledge would suggest that in general mothering ability improves with age to a point, could also possibly be a learned behavior and may be mediated by certain hormones.

### 8.3. Hormones and Neurotransmitters

In general, the maternal behaviors exhibited by mammalian species are associated with large shifts in key hormones which occurs across parturition [84]. Actions of hormones secreted from various sites such as the pituitary gland and peripheral endocrine tissues such as the gonads, placenta and adrenal gland interact with neural sites within the hypothalamus and central nervous system which may also be modulated by intrinsic neurochemical systems within the brain itself [95]. In most mammalian species, the periparturient period is characterized by an increase of plasma estradiol, prolactin and cortisol levels, and also by the activation of the oxytocinergic system during delivery of the neonate [96,97]. In cattle, more specifically, the normal hormonal milieu around calving consists of an increase in fetal plasma cortisol, maternal estradiol, prostaglandin and prolactin, with a decline in progesterone [98]. Previous work suggests that prior to, and post, calving, the immediate strong interest exhibited by the cow for newborn calves most likely initiated by the action of these hormones [2,3,92]. Hormonal changes during the birthing process are critical for maternal receptiveness, the let-down of milk and development of the cow-calf bond [23]. One hormone that has shown to be of particular importance for maternal behavior during the periparturient period is oxytocin. Oxytocin is a neuropeptide hormone secreted from the posterior pituitary gland of the brain and contributes to parturition in most mammals by influencing uterine contractility and secretion of milk via contraction of alveolar myoepthelium in the mammary gland [84]. Similar to most mammals, oxytocin in cattle may enhance positive social interactions such as maternal behavior, and facilitate the let-down of milk from the mammary gland which is essential to milk provision of the calf [94]. Interesting investigations have been performed in other species such as small ruminants and sheep that provide insight into the interactions of hormones such as oxytocin and maternal behavior. In sheep, oxytocin along with another steroidal hormone, estradiol, was important for stimulating maternal behavior as shown by intracerebroventricular injections of oxytocin which facilitated maternal responses in both pregnant and non-pregnant animals [99,100]. Central administration of oxytocin instigates the onset of maternal behavior in laboratory rats, proven by both models of direct intracerebroventricular infusion of oxytocin to the brain [101], and via oxytocin antagonists to the brain which inhibited observed maternal behavioral responses [102]. Another behavioral observational study in Grey Seals demonstrated that endogenous maternal plasma oxytocin levels were linked to mother-infant proximity [103]. This was reportedly the first study to link endogenous oxytocin concentrations in wild mammalian mothers with any type of maternal behavior and indicates a promising link. Although a first for wild mammals, which are similar to cattle housed extensively, other studies linking oxytocin and maternal behavior have been performed in intensively housed pigs [104] and primates [105]. The involvement of oxytocin in the formation of the maternal bond has been postulated to occur via recognition through olfactory cues, which through experimentation using sheep, it was found that the mRNA receptor expression and oxytocin reactivity increased in the olfactory bulb in sheep soon after parturition [106]. Preceding this interesting study, it was also found that maternal behavior which may be stimulated by endogenous oxytocin release through vaginal stimulation in sheep also required priming of other steroid hormones (estrogen and progesterone) to be successful [107]. Accordingly, higher oxytocin immunoreactivity and mRNA expression have also been reported in ewes primed with estrogen and progesterone [108]. The ovarian hormones of estrogen and progesterone during gestation therefore may act as the physiological primers for maternal behavior by acting centrally to promote the synthesis of oxytocin and its receptors in the brain. This study was conducted in periparturient and ovariectomized ewes, whereby immunohistochemistry was used on various brain regions to determine oxytocin immunoreactivity and gene expression. Breed-specific differences in ovine maternal behavior have at least partly been attributed to innate differences in hormonal physiology. For example, superior maternal behaviour of Scottish Blackface ewes over Suffolk ewes, observed at lambing (assayed via video recordings), has been correlated with higher higher estradiol and estradiol-to-progesterone concentration ratios during late gestation [87]. The onset of maternal behavior is therefore less likely influenced by a single hormone, and is more likely the consequence of several hormones acting in concert. This work is also important as it provides crucial insights into a likely interdependence between genetics, behavior and innate hormonal physiology. The previously identified hormonal cues recognised in sheep were also tested in cattle, and were found to be significantly complex. In a particular study, primiparous heifers were administered an epidural anesthetic to block the stimulation for endogenous oxytocin release. Priming of these heifers with estradiol or progesterone had no effect on the expression of maternal behaviors such as sniffing, licking and allowing suckling [92]. This work attempted to explore whether previously identified hormonal cues found in sheep and other lab animals where similar in cattle, however it was found that the same endocrine pathways did not appear adequate for induction of maternal behavior in heifers [92]. Despite these results in heifers, and given the promising findings in other species, it was no surprise that these links continued to be explored further in the bovine. Authors have attempted to investigate this using oxytocin, amongst other tests such as ocular thermal imaging and cortisol levels, as a physiological biomarker of maternal behavior. It was found that there was no statistically significant correlation between saliva oxytocin concentrations and maternal behavior, indicating its limited use as a suitable biomarker [2]. Therefore, the hormonal changes and interactions that instigate maternal behavior in the periparturient cow may warrant further investigation. Whilst numerous studies in other species have shown oxytocin alone may be linked to maternal behavior [101,103,104,105,106], in cattle and other ruminants, it seems to be more complex. 

Similar to the aforementioned oxytocinergic pathways, other major neurotransmitters explored include those affiliated with the dopaminergic system. Dopamine has been demonstrated as a crucial modulator of maternal behavior either through dopamine agonism [109,110] or antagonism [111,112,113] that may either promote or restore maternal care in rodents. Whilst no endocrinological profiling studies exist in cattle exploring direct relationships of dopamine to maternal behavior, authors have found interesting genetic links between generalised temperament tests and dopamine related genes [114].

Other neurotransmitters that have been explored in livestock, specifically sheep, include norepinephrine. Blocking of β-adrenergic receptors in the olfactory bulb in ewes will prevent olfactory learning of the offspring immediately after parturition [115]. In rodents, direct interruption of norepinephrine release through surgical transection of ascending noradrenergic pathways will interfere with certain aspects of behavior such as nest building and lactation [116]. Similar to norephinephrine, serotonin is another neurotransmitter partially implicated in the onset of maternal behavior. Using gene knockout techniques that control enzymatic production of serotonin, it has been shown that pup retrieval and maternal care may be reduced in mire [117]. Whilst there are some fairly well-established theories proven through experimentation involving the major gonadal hormones such as estradiol and progesterone as well as neurotransmitters including oxytocin, dopamine and norepinephrine, other hormones involved with stimulating maternal behavior, such as prolactin, have also been explored in other non-bovine species.

Prolactin is a polypeptide hormone that is mainly secreted by the lactotroph cells of the anterior pituitary gland, and has a significant involvement in a wide number of biological processes; which includes predominantly lactation, reproduction and other homeostatic processes [118,119,120]. Its secretion from the anterior pituitary is controlled by the hypothalamus, mostly via the action of dopamine, which acts antagonistically via the D2 subclass of dopamine receptors present in lactotrophs [119]. In general, yhe primary actions of this hormone, particularly in females, are associated with lactation, corpora lutea function in some species, and maternal behavior [121]. Endogenous levels of prolactin are known to significantly increase during pregnancy and late gestation, further illustrating its association with the pregnant animal [122]. As its name would suggest, prolactin has been possibly the most intensely researched hormone relating to mammary growth and lactation in dairy cattle, and there is extensive literature which has studied these effects [123]. However, the relationship between mothering ability and prolactin in cows has yet to be characterised. Past studies in other mammalian species have demonstrated that prolactin concentrations in the periparturient period is a crucial determinant of maternal behavior, and even more specifically that high prolactin during late gestation in mammals could ‘program’ offspring to become good mothers [122,124,125,126,127,128]. The association of prolactin and maternal behavior has been well characterized in humans [129], and other species [122,124,126,127], and is known to have a critical role in instigating postpartum maternal behavior in these species [130]. Inexperienced female rats that had their pituitaries removed (hypophysectomised) and then were administered injections of Ovine prolactin or had ectopic pituitary grafts with prolactin displayed complete maternal behaviours significantly faster than those without, defining an important role for this hormone in inducing pup retrieval and mothering. It was suggested that prolactin released by the pituitary gland feeds back on the central nervous system to affect the responsiveness of the female to young [125]. Similar results were found in hamsters which were exposed to a D2-receptor dopamine agonist (prolactin antagonist), bromocriptine. Hamsters exposed to treatment with bromocriptine showed significant disruption of maternal responsiveness, and a treatment group which was administered prolactin after bromocriptine administration showed restored behaviors [127]. It has also been concluded in rabbit models that prolactin acting prepartum may facilitate and maintain maternal behavior immediately postpartum and throughout lactation [126]. Other authors have also suggested that maternal exposure to prolactin during pregnancy is fundamental in stimulating the immediate onset of maternal behavior both during, and after parturition [125]. Elevations in endogenous prolactin levels during early pregnancy has also been hypothesized to be crucial for postpartum maternal behaviors [122]. In an experiment which artificially reduced endogenous prolactin levels during gestation, again using the dopamine agonist, bromocriptine, postpartum females showed a higher degree of anxiety compared to controls, and correspondingly, treatment with prolactin in early pregnancy reversed the anxiety seen in bromocriptine treated mice [131]. Interestingly, similar studies have found that bromocriptine rodents could also have restoration of maternal behaviors through administration of placental lactogen which both physiologically and structurally similar to prolactin [132]. This work also provides unique insight into the importance of placental hormones in controlling maternal behavior.

The intergenerational effects of prolactin on maternal behavior and subsequent fetal programming have been another area of investigation. The theory that a mother’s nurturing ability is regulated by certain factors present during her own fetal development in utero was tested [124]. Behavioral observations of arch-back nursing were used as a proxy for nurturing ability, and this was compared in mice exposed in utero to prolactin injections or saline injections. Interestingly, it was found that there were some notable differences in prolactin concentration in plasma and nurturing ability of the mothers. Furthermore, the neglect behaviors by the pre-programmed behaviorally deficient mothers could be rescued by administering prolactin itself during the latter half of pregnancy, similar to that reported by [131]. It was summarized that prolactin from the dam may activate certain neural circuits in the fetus that are required for the expression of certain maternal behavioral characteristics such as nurturing ability when fully mature [124]. However, more recent work by these same authors aimed to emulate this study by artificially lowering prolactin concentrations during pregnancy, and unfortunately did not find the same relationships [133]. Although different findings between the experiments are apparent, this work may still suggest a possible transgenerational effect associated with the concentration of prolactin that needs to be explored further. Genetics and transgenerational hormone profiling is discussed in the genetic section below.

Perhaps the closest studies which may provide insight into the effect of prolactin on behavior in the cow have been performed in sheep [86]. In a study of interest, the receptor for prolactin (PRLR) in euthanized sheep (via PLRR mRNA extraction) was measured and compared to maternal behavior recorded prior. Expression of PRLR mRNA was higher in ewes which displayed normal maternal behaviors [86]. Whilst this provides a promising insight into the effects of prolactin and behavior, a previous study which was performed in Australia delivered opposing findings. Ewes which were observed to desert their lambs at lambing were injected intramuscularly with 10–30 mg of prolactin within an hour of lambing. No changes in behavior were observed and prolactin injections also failed to overcome desertion behaviors [134]. These disagreeing findings exemplify the difficulties in attempting to establish that a link to maternal behaviour in livestock is fully attributable to the action of a single hormone. However, the study performed by [134], was performed some time ago and included a small sample size, and other studies have shown that the levels of prolactin prepartum, as opposed to postpartum appear to be more important for initiating the maternal response, as well as more sustained administration of prolactin if a behavior is to be rescued [124,125].

Prolactin has also been investigated as a candidate gene for productivity traits in Angus cattle, and it was found that through the genomic coding of the prolactin gene, the prolactin haplotype was highly corelated to production traits such as pre-breeding body condition (BCS) and weight, calving date, calf birth weight, cow body condition at weaning and 205 day calf weight [135]. It was summarised that cows with the prolactin haplotype may have the greatest cow efficiency, potentially suggesting promising links between this hormone and maternal success in the breeder cow [135]. Whilst there is already a well-established association between prolactin secretion and milk production [123,136,137], there appears to be little research which has investigated the link between endogenous concentrations of prolactin in the cow, maternal behavior and calf performance. Whilst prolactin has been heavily investigated in relation to lactation and mammogenesis, its predominant role in inducing mammogenesis also remains controversial, although at this point in time most authors would agree prolactin is one of the primary drivers, along with other hormones such as growth hormone, placental lactogen, progesterone, and estradiol [123]. It would therefore seem plausible that higher levels of prolactin would be found in cows that produced more milk as shown by [138]. Additionally, work has shown that cows suckling twin calves had higher levels of milk prolactin concentrations [139]. The previously established role in prolactin in lactogenesis, as well as the relationship between prolactin and cow efficiency indeed provides an indication for further research in this field. In summary, the array of studies performed in multiple species suggest that there could be plausible uses in hormonal profiling to investigate relationships between cattle and superior mothering ability. Additionally, the investigations linking possible breed differences and innate hormonal physiology, such as those performed in sheep [87], could identify that there is a key link between a combination of host genetics, hormones and maternal behavior.

## 9. Influence of Genetics on Maternal Behavior

Maternal behavior in cattle has been reported to be at least, in part, genetically controlled [3]. More broadly, in a review of the molecular and genetic bases of maternal behavior in mammals by [84], it has been suggested that the learned maternal behavior of the mother from her early experiences are indirectly related, and potentially moderated by the mothers’ own genetic profile and epigenetic mechanisms. Whilst this review mainly focused on laboratory animals like rats, it provides a unique insight into some of the more intricate factors influencing mothering ability that are yet to be studied in livestock. This review surmised that host genetics is associated with behavior via three key hormonal pathways involving oxytocin, dopamine and 5 HT (serotonin) [84]. The underlying genetic and epigenetic mechanisms contributing to some of these psychoneurobiological mechanisms associated with behavior were explored at length. Whilst this review focused mostly on humans and rats, it was concluded that the mother’s innate genetic profile moderates how behavior is expressed, and it is likely that this could be similarly reflected in livestock. This review also highlighted other relevant insights in humans which demonstrate a genetic link to maternal behavior through the action of hormones such as oxytocin. In a human study individuals had blood sampled for oxytocin and a mouthwash for salivary DNA, as well having interactions between parent and infant observed [85]. Of the individuals who were genotyped for the oxytocin receptor (*OXTR*) and *CD38* (an ectoenzyme that mediates the release of oxytocin from the brain) risk alleles, both the levels of plasma oxytocin levels and parental interaction were lower. This suggests a possible link between certain gene pathways, hormone levels, particularly oxytocin, and maternal behavior. Similar studies have also investigated this genetic-hormonal pathway with prolactin. Pup-directed maternal behavior has been studied using mice typed for the Prolactin Receptor (PRLR) mutation gene. Using mice that were either pregnant or exposed to pups, behavioral observations based on pup retrieval and crouching were made, and it was found that both homozygous and heterozygous PRLR mutant females showed a deficiency in maternal behavior and care [140]. Given the promising findings in lab animals, the concept was further explored in sheep [86]. Prolactin Receptor (PRLR) mRNA was extracted from the pituitary region of slaughtered ewes and analyzed by qPCR. Prior to euthanasia, four main maternal behaviors were observed including lamb grooming, acceptance and suckling (normal maternal behavior); as well as aggression and suckling rejection (abnormal maternal behavior). The expression of higher PRLR mRNA was found in the ewe group with ‘normal maternal behavior’ [86]. The action and effectiveness of prolactin is unsurprisingly mediated via its hormone receptor (PRLR) in the brain, and therefore work of this kind provides unique insight into receptor characteristics of certain hormones that could be carried across generations. Inferences from other species provide insight into the transgenerational links associated with maternal behaviour. It has been suggested that transgenerational programming of maternal behavior could be triggered by exposure to certain stressors prenatally in lab rats [141]. This was explored through experimentation. Rats were exposed to daily stress treatments of a restraint container and swim stress throughout the latter half of gestation [141]. The impact of stress treatments was measured using observations of ‘tail chasing’ behavior. The mothers exposed to effective stressors, passed on behavioral responses to her offspring and this impacted mothering ability across generations. These findings were also consistent with a previous study by [142] who found that rats exposed to stressors during gestation altered postpartum maternal care behaviors in the offspring. This would suggest that the intergenerational effects are slightly more complex than being attributed to things such as single hormone or experience, and nonetheless warrant further investigation. There are no known studies that have been published on the transgenerational hormonal effects in livestock, and more specifically, these models are also yet to be characterized in cattle.

Potentially selectable maternal traits are of high economic importance to producers, and at this point in time it would seem there is lack of efficient selection tools to improve such traits as maternal behavior [3]. Estimated breeding values (EBVs) have been used in livestock for this, however they have previously only focussed on ‘Maternal Ability’ which is calculated based on performance figures such as eight-week weight of lambs [143], as opposed to behavior directly. The usefulness of a Maternal Behaviour Score (MBS) for selection in sheep has had different opinions. It was found that simply using a Maternal Behavior Score (MBS) based on a ewe’s reaction to infant handling, had low-moderate heritability in one study [144]. However, other studies have differed, finding a moderate heritability and high degree of repeatability when using the MBS score to aid in genetic selection [145]. In beef cattle specifically there are significant differences in reported heritability of Maternal Behavior (MB) in the literature, although in general the repeatability of MB based on an MBS has low-moderate heritability making it difficult as a selectable trait [3]. Additionally, in this study, very few gene quantitative trait loci (QTL) were detected for MB, with the main QTL region for MB being chromosome six, which explained only 18.8% of the estimated total accuracy of genomic prediction for MB [3]. Other indirect EBVs for maternal ability also exist in Australian cattle production, such as 200 Day Milk which correlates with increased milk production and the maternal contribution to weaner weights [146]. This would suggest that there is a need to further investigate suitable biomarkers and measurable phenotypes for maternal behavior in the cow which could be possibly used for selection of heritable traits in a production setting.

## 10. Calf Factors

Calf factors are also related to differences in the expression of maternal behavior in cattle. Some of the these calf factors include sex and weight [2]. Calf weaning weight was also found to be associated with cow-calf proximity in a behavioral observation study which followed 10 bull calves and 10 heifer calves until weaning. It was found that cows and calves spent more time together when the calf was a female (compared to male), and more time spent with calves which had a lower weaning weight, which seems somewhat counterintuitive [25]. However, some disagreements were found by others, for instance when observing cows from day three to 30 into lactation, it was found that male calves received both more maternal protection and more frequent nursing compared to females [80]. The increased interaction with male calves was also found using Zebu *Bos indicus* animals as a comparison, and they found that the increased frequency of interaction between cows and male calves was also independent to the sex of their own calf [147]. Cows in better body condition have also been shown to provide more intense protection of their young, supported by a clear relationship between cow body condition and calf weight gain and nursing [80]. In general, the relationships between calf weight, weaning weight and cow body condition seem plausible, although the direct correlation of these factors with maternal behavior is likely more complex and multifactorial.

## 11. Mustering and Handling

Mustering is a term used in Australia to describe the process of gathering/rounding up groups of cattle from paddocks into yarding facilities for processing and handling [148]. Mismothering has been found to be associated with mustering and handling in northern Australian settings likely due to interfering with the early cow calf bond [149], and has historically been estimated to result in approximately 4% of calf losses [150]. Mustering is a term used in Australia for gathering cattle from large paddocks and moving them into yarding facilities for processing, which often occurs once-twice yearly on large, extensive operations in the tropics [90]. A review suggested that mustering of young calves could indeed be detrimental however, the direct quantifiable effects of mustering practices on overall calf mortality rates in cattle reared under extensive Australian conditions are unknown at this point [90]. Nonetheless, mustering around the time of calving and also low mustering efficiency are recognised risk factors that are highly likely to affect milk delivery and therefore calf survival [151]. The effect of human interference on the formation of the early cow-calf has also been previously investigated using motivation tests. It was found that early cow-calf separation prevents much of the cow’s maternal behavior, and the motivation for this contact is greater in cows that had time to be suckled by their calf [152]. This was demonstrated via motivation tests involving a one-way push gate in cows that were: (i) separated from their calf within two hours after birth, (ii) allowed to spend nights with their calf but had an udder net; (iii) allowed to spend nights with their calf and were allowed to be suckled. Group (iii) showed the greatest motivation to re-join their own calves [152]. The early postpartum period in the cow, known as the sensitive period, is particularly susceptible to human interference and can hence hinder the development of the cow-calf bond [153]. The response of the cow to human interference during the early postpartum period varies according to breed type and production system, which may suggest that certain breeds that have had different levels of historic human intervention will respond differently to human interference [23,154].

## 12. Assessment of Maternal Behavior

**Figure 4:**
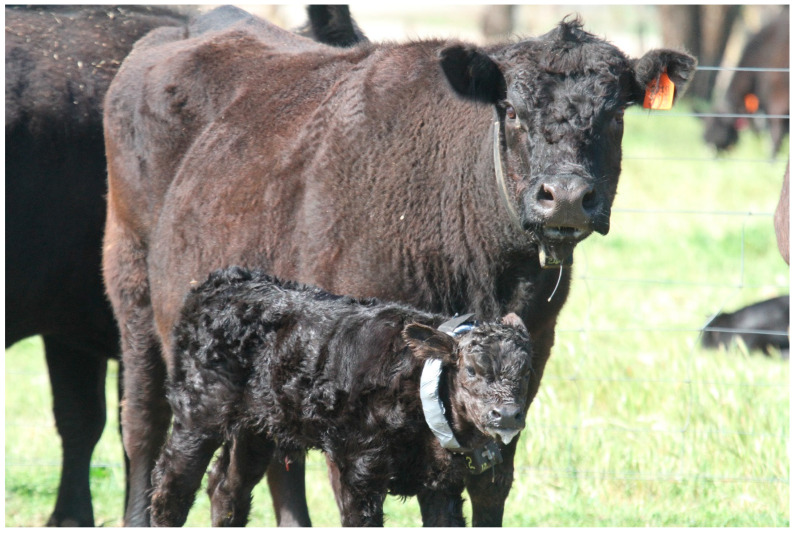
Mature Angus beef cow and her calf with deployed proximity logger collars.

A key challenge associated with behavioral traits is that they are often difficult to measure and quantify. Recording maternal behavior on a large scale in extensive production systems has historically been impractical, particularly when attempting to define an individual phenotype [23]. Therefore, there is a need to explore alternative traits that are easily measured and could be used as indicators of maternal behavior. Studies performed in beef and sheep cattle which aim to predict or record maternal behavior typically include a mix of mostly observational qualitative studies—which involve either scoring systems or subjective assessments [2,13,25,39,47,49,50,51,52,82], and a few quantitative measurement studies involving beef cows typically under extensive settings using remote monitoring devices [21,38,40,43,44]. Table 1 (page 6) provides a summary of some of the studies in which maternal behaviour specifically has been recorded. In the observational studies used previously for ruminants, a Maternal Behavior Score (MBS) using an arbitrary scale has occasionally been applied and is based on the cow and/or ewe’s response to handling of the newborn by stockpersons [2,47,48,51]. Typically a higher MBS score delineates more aggression exhibited by the cow toward human handling of her calf [47]. Previous work has found variable findings when using these scores to investigate links into calf performance and therefore raises questions as to the relationship between cow aggression and successful rearing of a calf [64]. Whilst observational studies may provide useful information, potential shortfalls of this work include being able to obtain unbiased, quantitative and objective data that can be consistently measured by different researchers across different generations of cattle. With recent investments in automated sensing devices, it has become practical to objectively and accurately measure social behavior amongst cattle reared in extensive systems and environments more objectively. Proximity loggers have been widely used in domestic and wild species to study and quantify patterns of intra- and inter-species interactions relating to behavioral, ecological and evolutionary questions [44,45,155]. Prior to the use of these devices, social behavior was primarily assessed via observational studies, highlighting objective and quantitative limitations in data interpretation. Proximity loggers are remote monitoring devices which automatically collect animal interaction data using user-defined parameters such as detection distance and separation time. These devices are attached to animals using neck collars, harnesses or ear tags and they record frequency and duration of contacts when tagged animals come in a pre-set distance of one another. See Figure 4 of deployed proximity logger collars on a cow-calf pair.

These commercially available systems have been previously validated to be accurate and reliable in assessment of various animal behavioral patterns [44,156,157,158]. Specifically in the Australian context, some studies have used contact-based telemetry to explore animal affiliations in beef cattle and calves with varying degrees of success in [21,38,40,45]. In these studies, proximity logger collars (Sirtrack Ltd. Havelock North, New Zealand, now Lotek) were fitted to cows and calves to measure interactions over time. The devices themselves emit and receive UHF waves and are programmed according to a predetermined read range (coefficient range: 0–62) and separation time (range: 1–255 s) to receive and log contacts [38,40]. These collars work by recording an interaction when two collared animals come within a predefined detection range and cease recording after failing to reach a signal following predefined separation time. The devices weigh approximately 500 g and are attached to the animal with a quick-release collar made of fabric material. When fitted, the collars are checked to make sure they are tightened appropriately. The devices themselves provide no risk to the animal on which they are deployed, and in the studies in which they have been used, no detrimental side effects or animal welfare considerations have been reported [159]. Each logger records the identification of the other, and the date and start time of the encounter is recorded by both loggers and stored on the memory [38]. When the primary logger (receiver) fails to receive the signal at a defined ‘separation time’, (which can range from 1–255 s, average 30 s) the duration of the encounter is finished and recorded [38,40]. These studies used different pre-defined read ranges between collars, which was set to either 4 m [38], 5 m [45], or 7 m [40] to record a close encounter. Loggers were able to record duration and number of interactions within this set distance. One potential shortfall noted through the use of proximity-logger data was that absolute spatial precision was not possible as the UHF radio waves can be reflected and/or absorbed through naturally occurring obstacles/compounds [40]. Furthermore, when comparing the use of tri-axial accelerometers, UHF proximity logger collars and visual observations, it was found that not all close encounters recorded during the suckling events were captured by the proximity loggers, indicating that proximity loggers could not be used in lieu of accelerometers to record suckling bouts in cow-calf pairs [38]. This was likely attributed to interruptions in the way that UHF radio waves were transmitted and received by being absorbed, refracted and reflected by objects in the study area, including the body of the animals wearing the collars [38]. These findings suggest that although UHF and/or GPS collars may provide useful information about animal movements and spatial patterns, there may be limitations in comprehensively recording all of the close, more intimate encounters between cow and calf. The strength of the cow-calf bond after calving is also reinforced through suckling events, and an important aspect of mothering and milk provisioning for the newborn [40]. Additionally, the critical time for transfer of immunity via colostrum to the calf is within the first few hours of life [73,160,161], which is also at a time when cows may reportedly ‘stow’ their calves in a secluded location whilst they graze [162]. Therefore, contact alone may not be fully reflective of normal maternal behaviors of the cow in the days immediately after calving. Despite these findings, researchers would still agree that interactions recorded via UHF proximity loggers do otherwise provide valuable information for measuring social affiliations between the cow and calf [40,45]. Other important considerations associated with the use of proximity loggers relate to the existence of a moderate degree of intrinsic inter-logger variation [155]. Therefore, loggers must be calibrated prior to use, along with observational data, to make any necessary adjustments over time [21,43,45,158]. Whilst remote monitoring technologies such as UHF proximity loggers, GPS systems, and activity meters are very applicable tools in extensive grazing scenarios, a shortfall in these devices is that only certain aspects of behavior are being recorded, that being contact and activity. Maternal behavior in the cow is complex, and a single behavioral pattern that can be readily assessed to approximate overall behavior is unlikely to exist at this time. Ideally, research that involves the use of multiple recording devices, and maternal behavior scores (MBS), alongside observational data that can be comparable to calf performance measures may provide even more insight into identifying superior cows.

## 13. Conclusions

Bovine maternal behavior is a complex phenotype that can be difficult to measure and predict objectively. The effect of age, breed, parity and husbandry practices on behavior has been reported in the literature at length and is known to vary significantly according to these factors. Despite previous attempts, at present, no known heritable and repeatable biomarkers for superior maternal behavior exist in the cow [2]. Whether this is attributable to inaccuracies in current hormone assays or unreliable estimates of good maternal instinct requires further investigation. Additionally, maternal over-vigilance or aggression could indeed prove counter-productive in certain instances, and calf performance may indeed be a more suitable means to assess the superior rearing of a calf by a cow. A significant challenge, particularly in extensive beef production systems, is associated with objectively and quantitatively assessing maternal behavior. Previous studies have validated the use of contact-based telemetry and/or activity metres to remotely assess behavioral traits in extensive production settings and this may hold promise for future research. The use of maternal behavior scores in response to calf handling have been used in observational studies and compared to calf production traits such as average daily gain and weaning weights. However, past studies have reported mixed findings associated with the heritability and repeatability of observational scoring when trying to establish a link between selectable traits across generations. Work in other species, namely small mammals and rodents, has shown promising links between hormones like prolactin and maternal behavior. Further exploration into the endogenous levels of hormones around calving in cows and maternal behavior could provide valuable insights. A key challenge going forward for the livestock industry will be to find an accurate biomarker that is easily measurable and predictable across generations. Endocrinological profiling may hold promise in future for this purpose, and further research is indeed indicated. Previous work which explores the interplay between genetics, hormonal physiology and maternal behavior provides crucial insights into being able to find these links, and further research is needed in cattle. Ultimately, the complexities in measuring behavioral traits in livestock, as well as the innate variation in hormone levels between individual animals may account for the lack of success when looking to establish a heritable, repeatable, and low-cost assay that may be used to identify superior mothers in the bovine species.

## Figures and Tables

**Table 1:** Summary of methods and techniques used to investigate maternal behavior in ruminant livestock.

Measurement/Technique	Reference	Species/Breed
Observational Study	[13]	*Bos indicus*
Proximity logger contact data	[38]	Belmont Red, Brahman
Proximity logger contact data; accelerometers	[43]	Belmont Red, Brahman
Proximity logger contact data	[40]	*Bos indicus*
Proximity loggers, GPS collars	[44]	*Bos indicus*
Proximity loggers and Observational	[21]	Brahman/Droughtmaster ×
Proximity loggers	[45]	Brahman/Droughtmaster ×/Belmont Red
Proximity loggers	[46]	Merino Ewes
Observational Maternal Behavior score	[47]	Beef cattle various breeds
Observational Maternal Behavior Score	[48]	Sheep various breeds
Observational study	[2]	Beef (Simmental), Dairy (German Black Pied)
Observational study	[49]	Dairy cows
Observational study	[50]	Sheep (Scottish Blackface and Suffolk)
Observational study	[25]	Beef cattle
Observational study	[39]	Wild Maremma beef cattle
Observational study	[51]	Angus and Simmental cattle
Observational study	[52]	Nelore and Guzerat cattle
Observational study	[53]	Friesian (Dairy), Saler (Beef)
Video recording & Observational	[54]	*Bos indicus*, *Bos indicus* × *Bos taurus*

## Data Availability

All data or further images are available on request.

## Appendix A

Misadventure is the term used for when either the dam or offspring become separated and/or lost usually due to environmental factors (for instance calf wanders and gets displaced on the wrong side of a fence) [163].
